# The Human Adenovirus Type 5 E4orf6/E1B55K E3 Ubiquitin Ligase Complex Enhances E1A Functional Activity

**DOI:** 10.1128/mSphere.00015-15

**Published:** 2015-11-11

**Authors:** Frédéric Dallaire, Sabrina Schreiner, G. Eric Blair, Thomas Dobner, Philip E. Branton, Paola Blanchette

**Affiliations:** a Department of Biochemistry, McGill University, Montreal, Québec, Canada; b Institute of Virology, Technische Universität München/Helmholtz Zentrum München, Munich, Germany; c School of Molecular and Cellular Biology, University of Leeds, Leeds, United Kingdom; d Heinrich Pette Institute, Leibniz Institute for Experimental Virology, Hamburg, Germany; e Department of Oncology, McGill University, Montreal, Québec, Canada; f The Goodman Cancer Research Centre, McGill University, Montreal, Québec, Canada; University of Michigan

**Keywords:** adenovirus, ubiquitin ligase, E4orf6, E1B55K, E1A

## Abstract

Human adenovirus (Ad) E1A proteins have long been known as the central regulators of virus infection as well as the major source of adenovirus oncogenic potential. Not only do they activate expression of other early viral genes, they make viral replication possible in terminally differentiated cells, at least in part, by binding to the retinoblastoma (Rb) tumor suppressor family of proteins to activate E2F transcription factors and thus viral and cellular DNA synthesis. We demonstrate in an accompanying article (F. Dallaire et al., mSphere 1:00014-15, 2016) that the human adenovirus E3 ubiquitin ligase complex formed by the E4orf6 and E1B55K proteins is able to mimic E1A activation of E2F transactivation factors. Acting alone in the absence of E1A, the Ad5 E4orf6 protein in complex with E1B55K was shown to bind E2F, disrupt E2F/Rb complexes, and induce hyperphosphorylation of Rb, leading to induction of viral and cellular DNA synthesis, as well as stimulation of early and late viral gene expression and production of viral progeny. While these activities were significantly lower than those exhibited by E1A, we report here that this ligase complex appeared to enhance E1A activity in two ways. First, the E4orf6/E1B55K complex was shown to stabilize E1A proteins, leading to higher levels in infected cells. Second, the complex was demonstrated to enhance the activation of E2F by E1A products. These findings indicated a new role of the E4orf6/E1B55K ligase complex in promoting adenovirus replication.

**IMPORTANCE** Following our demonstration that adenovirus E3 ubiquitin ligase formed by the viral E4orf6 and E1B55K proteins is able to mimic the activation of E2F by E1A, we conducted a series of studies to determine if this complex might also promote the ability of E1A to do so. We found that the complex both significantly stabilizes E1A proteins and also enhances their ability to activate E2F. This finding is of significance because it represents an entirely new function for the ligase in regulating adenovirus replication by enhancing the action of E1A products.

## Abstract (precis)

Following our demonstration that adenovirus E3 ubiquitin ligase formed by the viral E4orf6 and E1B55K proteins is able to mimic the activation of E2F by E1A, we conducted a series of studies to determine if this complex might also promote the ability of E1A to do so. We found that the complex both significantly stabilizes E1A proteins and also enhances their ability to activate E2F. This finding is of significance because it represents an entirely new function for the ligase in regulating adenovirus replication by enhancing the action of E1A products.

## INTRODUCTION

The major regulators of human adenovirus replication are the products of early region 1A (E1A), which encodes a series of species, notably products of the 13S (289R) and 12S (243R) E1 mRNAs (1). Both interact with the retinoblastoma (Rb) protein and other members of the “pocket” protein family that regulate E2F transcription factors through complex formation with transcriptional repression complexes (2, 3). Binding by E1A products frees E2F to induce expression of both early viral transcripts and cell cycle genes that regulate the initiation of DNA synthesis, thus permitting replication even in terminally differentiated epithelial cells, the major targets of human adenoviruses (Ads) (4–6). In addition, E1A 289R contains a transcriptional activation domain that promotes transcription of early viral genes (7). Thus, E1A products act early after infection to activate expression of viral genes required for replication and to promote conditions for effective viral DNA synthesis. Both the 13S/289R and 12S/243R E1A proteins are known to exhibit very short half-lives in infected cells (8, 9). Two regions of E1A proteins contribute to this property: one near the N terminus, which interacts with 19S proteasomal proteins (10, 11), and PEST sequences in the C terminus, in which phosphorylation is crucial to target E1A products to proteasomes (10).

The early adenovirus E4orf6 and E1B55K products have been shown in all human adenovirus species to form functional cullin-based E3 ubiquitin ligase complexes that enhance viral replication by degrading a number of cellular proteins (12–14). The E4orf6 protein associates via multiple BC boxes with cellular elongins B and C to facilitate binding of either Cul5 or Cul2 and other components to form the core ligase complex (13, 15–18). E1B55K associates with E4orf6 only when present in the ligase complex, as E4orf6 BC box mutants do not interact with E1B55K (17). E1B55K is believed to function as the major substrate acquisition component of the ligase complex. Although a number of substrates of the ligase have already been identified (17–30), additional functions could exist that enhance viral replication.

In an accompanying article (31), we demonstrate that when expressed at high levels, the E4orf6/E1B55K complex mimics E1A activation of E2F transcription factors by inducing hyperphosphorylation of Rb and disrupting the Rb/E2F complex. Here we show that this complex both significantly stabilizes E1A proteins and appears to enhance the ability of E1A products to disrupt E2F/Rb complexes and activate E2F. These findings suggest a new and important function for the ligase complex in adenovirus infection.

## RESULTS

### The E4orf6/E1B55K complex mimics E1A functions even at low levels typical of virus-infected cells.

**FIG 1:**
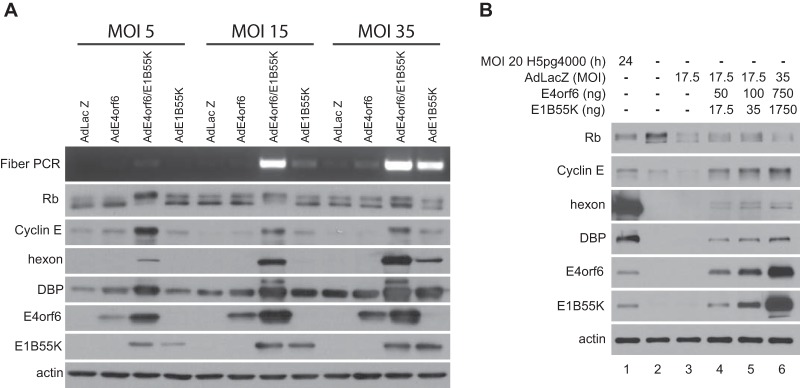
Effects of E4orf6 and E1B55K at low-level expression. (A) H1299 cells were infected for 48 h with the indicated viral vector at the indicated MOIs and complemented with AdLacZ for an even total of virus particles for each set. Whole-cell extracts were immunoblotted as indicated using the appropriate antibodies, and PCR for the fiber gene was performed. (B) H1299 cells were transfected with increasing amounts of cDNAs expressing E4orf6 and E1B55K and infected with AdLacZ at the indicated MOI for 48 h or infected with wild-type virus at an MOI of 20 for 24 h. Whole-cell extracts were immunoblotted as indicated using the appropriate antibodies.

In our previous studies, described in reference 31, we reported that, when expressed at high levels in the absence of E1A products, the Ad5 E4orf6/E1B55K ligase complex could induce the hyperphosphorylation of Rb, disrupt Rb/E2F complexes, and activate E2F-dependent transcription. These effects were more modest than those induced by E1A. Nevertheless they appeared to be dependent on the formation of the E4orf6/E1B55K ligase complex and may result from an interaction of E4orf6 proteins with E2F transcription factors. One concern in these studies was that although the E4orf6/E1B55K complex clearly was able to mimic E1A functions related to activation of E2F, with resulting effects on DNA synthesis and virus production, it was possible that these effects only occur when E4orf6 and E1B55K are expressed at high levels, far in excess of those routinely detected in wild-type virus-infected cells. To address this issue, two types of study were conducted. Experiments in our previous studies were performed with infection by viral vectors at a multiplicity of infection (MOI) of 35, as done previously in our screen for ligase substrates (22, 32). To determine if expression of E4orf6 and E1B55K induced these effects at lower levels, H1299 cells were infected with AdE4orf6 and AdE1B55K at MOIs of 5, 15, and 35. As shown in Fig. 1A, reduced levels of E4orf6 and E1B55K were seen at lower MOIs, but nevertheless significant induction of viral DNA synthesis, hyperphosphorylation of Rb, and cyclin E, hexon, and DNA binding protein (DBP) levels were apparent, even at an MOI of 5 although the effects were somewhat reduced. To address this issue further, studies were conducted in which we attempted to match directly the levels of E4orf6 and E1B55K present in cells infected by wild-type Ad5 at an MOI of 20. Cells were infected with AdLacZ vector and cotransfected with increasing amounts of plasmid DNAs expressing either E4orf6 or E1B55K. Figure 1B shows that the levels of these products in wild-type-infected cells at 24 h postinfection (p.i.) (lane 1) were comparable to those in AdLacZ-infected cells transfected by the smallest amounts of plasmid DNAs (lane 4) in which significant hyperphosphorylation of Rb and increased cyclin E, hexon, and DBP levels were apparent relative to mock-infected (lane 2) and AdLacZ-infected (lane 3) cells. While the outcome, as in previous studies, was much less than with wild-type virus, these results indicated clearly that the complex can elicit its effects even at levels of E4orf6 and E1B55K normally present in virus-infected cells.

### E4orf6 increases the stability of E1A products.

**FIG 2:**
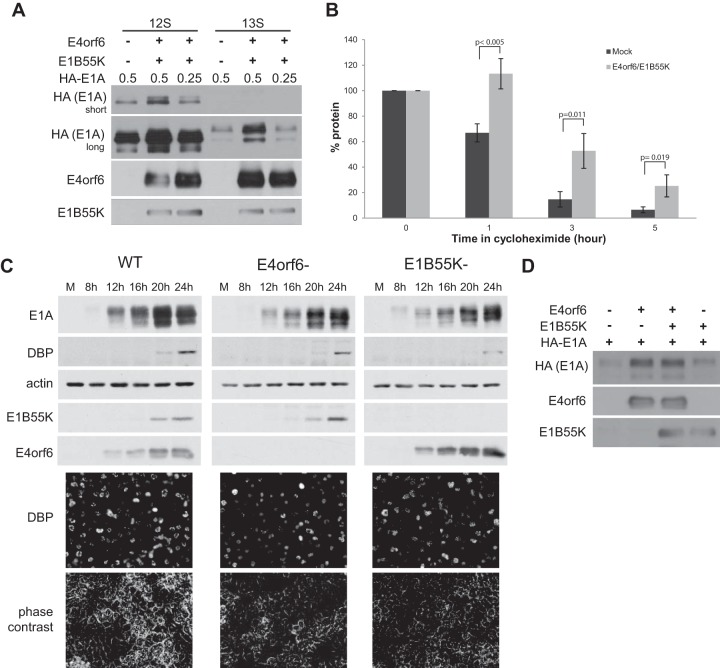
E4orf6/E1B55K increases the stability of E1A. (A) H1299 cells were transfected with plasmid DNA expressing E4orf6, E1B55K, and various amounts of E1A as indicated for 48 h. Whole-cell extracts were immunoblotted as indicated using the appropriate antibodies. (B) H1299 cells were transfected with plasmid DNAs expressing E4orf6, E1B55K, and 13S E1A for 43 h followed by 0 to 5 h after addition of 25 μg/ml cycloheximide. Whole-cell extracts were immunoblotted using E1A antibodies, and the blots were scanned and quantified with ImageJ. Each condition was normalized to its time zero (average of three experiments). (C) H1299 cells were infected with the indicated viruses at an MOI of 5 and harvested at the indicated times. Whole-cell extracts were immunoblotted as indicated using the appropriate antibodies. WT virus, H5*pg*4100; E4orf6-deleted, H5*pm*4154; E1B55K-deleted, H5*pm*4149. (D) H1299 cells were transfected with plasmid DNAs expressing E4orf6, E1B55K, and E1A as indicated for 48 h. Whole-cell extracts were immunoblotted as indicated using the appropriate antibodies.

E1A plays an important essential role in activating E2F to promote efficient viral replication and induction of viral and cellular DNA synthesis (33–36). Given our recent findings, we wondered what effect E4orf6/E1B55K might have in a context where E1A was also present. Both the 13S/289R and 12S/243R E1A proteins are known to exhibit very short half-lives in infected cells (8, 9), and thus changes in their synthesis or stability could affect the infectious cycle. To determine if the E4orf6/E1B55K complex affects E1A protein levels, plasmid DNAs expressing either the 12S or 13S E1A products were transfected into H1299 cells either alone or along with those expressing E4orf6 and E1B55K. Figure 2A shows that in both cases, expression of E4orf6/E1B55K significantly increased the levels of E1A products. This finding was somewhat surprising because, as discussed above, the viral ligase normally acts to degrade a variety of substrates. (It was also evident in Fig. 2A that by reducing the amount of E1A-expressing plasmid DNAs, levels similar to those seen with E1A alone could be achieved in the presence of E4orf6/E1B55K. This manipulation will be of importance in experiments described below in which we attempt to measure the effects of the E4orf6/E1B55K complex on E1A functions.) One obvious explanation for these increased E1A levels could be that E1A proteins are more stable in the presence of the E4orf6/E1B55K complex. To examine this possibility, cells were transfected with plasmid DNAs expressing 13S/289R E1A protein in the presence or absence of those encoding E4orf6/E1B55K, and after 43 h, cycloheximide was added for increasing times and the levels of E1A protein were measured by quantifying scans of E1A Western blots. Figure 2B shows the amounts of 13S E1A protein at various times following cycloheximide treatment. From these data, the half-life values were calculated, indicating that expression of E4orf6/E1B55K significantly increased the E1A half-life from 2.09 h in the control to 3.56 h, an effect consistent with the observed increased E1A levels shown in Fig. 2A. Similar results were seen with 12S E1A (data not shown). To determine if E4orf6 and E1B55K also affect the stability of E1A proteins in the context of an infection, H1299 cells were infected with wild-type virus, as well as mutant viruses in which the E4orf6 or E1B55K genes were deleted. Cells were lysed at 4-h intervals, and E1A, DBP, E4orf6, and E1B55K protein expression levels were examined by Western blotting with actin as a control. Figure 2C shows that deletion of the E4orf6 or E1B55K genes caused a reduction in the levels of E1A present, an effect not seen with another E4 mutant lacking E4orf4 (data not shown). Reduction of E1A levels was not due to differences in the proportion of infected cells in the population as analysis by immunofluorescence microscopy using anti-DBP antibodies indicated a similar percentage of positive cells in all cultures (Fig. 2C, bottom). Thus, these findings were consistent with a role for E4orf6 and E1B55K in stabilizing E1A proteins. Interestingly, Fig. 2C shows that deletion of E4orf6 or E1B55K resulted in some reduction of DBP levels, consistent with the effect that the E4orf6/E1B55K complex had in inducing the E2E promoter (31). To determine if both E4orf6 and E1B55K function to stabilize E1A proteins, H1299 cells were transfected with plasmid DNAs expressing HA-E1A, E4orf6, or E1B55K, and extracts were analyzed by Western blotting. Figure 2D shows that E4orf6 alone could induce a significant increase in E1A protein, whereas E1B55K alone exhibited only a small increase. These results suggested that E4orf6 might play a greater role in the stabilization of E1A products.

**FIG 3:**
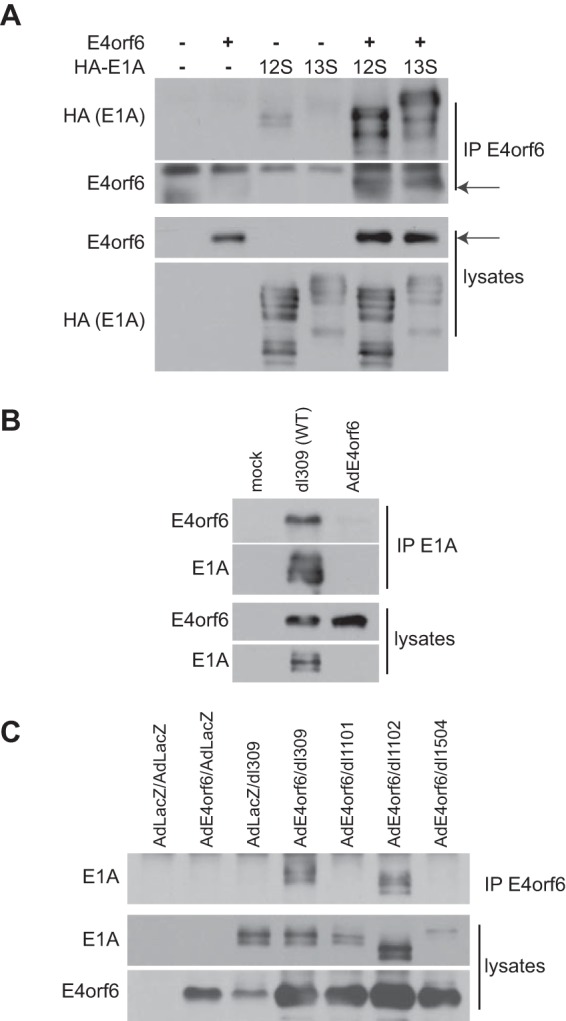
E4orf6 binds at the N terminus of E1A proteins. (A) H1299 cells were transfected with plasmid DNAs expressing E4orf6, 12S E1A, and 13S E1A proteins as indicated, and at 48 h, immunoprecipitates were obtained using E4orf6 antibodies as well as whole-cell extracts were immunoblotted as indicated using appropriate antibodies. (B) H1299 cells were infected with the indicated WT virus or negative-control AdE4orf6 viral vector for 24 h at MOIs of 20 and 5, respectively, to obtain similar expression levels of E4orf6 protein. Immunoprecipitates obtained using antibodies against E1A proteins as well as whole-cell extracts were immunoblotted as indicated using the appropriate antibodies. (C) H1299 cells were infected as indicated with WT virus (*dl*309) or E1A *dl*1101 (Δ4-25), *dl*1102 (Δ26-35), or *dl*1504 (Δ1-14) mutant viruses at an MOI of 20 FFU/cell and coinfected with AdE4orf6 or AdLacZ viral vectors at an MOI of 5 for 24 h. Immunoprecipitates obtained using E4orf6 antibodies as well as whole-cell extracts were immunoblotted as indicated using the appropriate antibodies.

To explain this effect on E1A stability, we determined if E4orf6 interacts with E1A products. Figure 3A shows that E4orf6 interacted with multiple forms of both 13S and 12S E1A products. No such interaction was observed in similar studies with E1B55K alone (data not shown). This interaction between E1A and E4orf6 proteins was also detected during virus infection. H1299 cells were infected with wild-type *dl*309 virus or the AdE4orf6 vector expressing E4orf6 as a negative control for the expression of E1A, and lysates were immunoprecipitated using anti-E1A antibodies. Figure 3B shows that the E4orf6-E1A interaction also was evident during virus infection. These results suggested that interactions between E1A products and E4orf6 might be responsible for increases in E1A stability. Although we have not conducted an extensive analysis of the E4orf6-E1A protein interactions, Fig. 3C shows studies using E1A mutants lacking various regions toward the N terminus. The results indicated that binding to E1A products requires a region at the N terminus between residues 1 and 25 (the *dl*1101 and *dl*1504 mutants) but not residues 26 to 35 (the *dl*1102 mutant). This N-terminal region corresponded to residues shown previously to be involved with interactions with 19S proteasomal proteins (10, 11). Thus, binding of E4orf6 might enhance E1A stability by inhibiting such interactions to slow degradation by the proteasome.

### E4orf6 and E1B55K enhance E1A effects on E2F.

**FIG 4:**
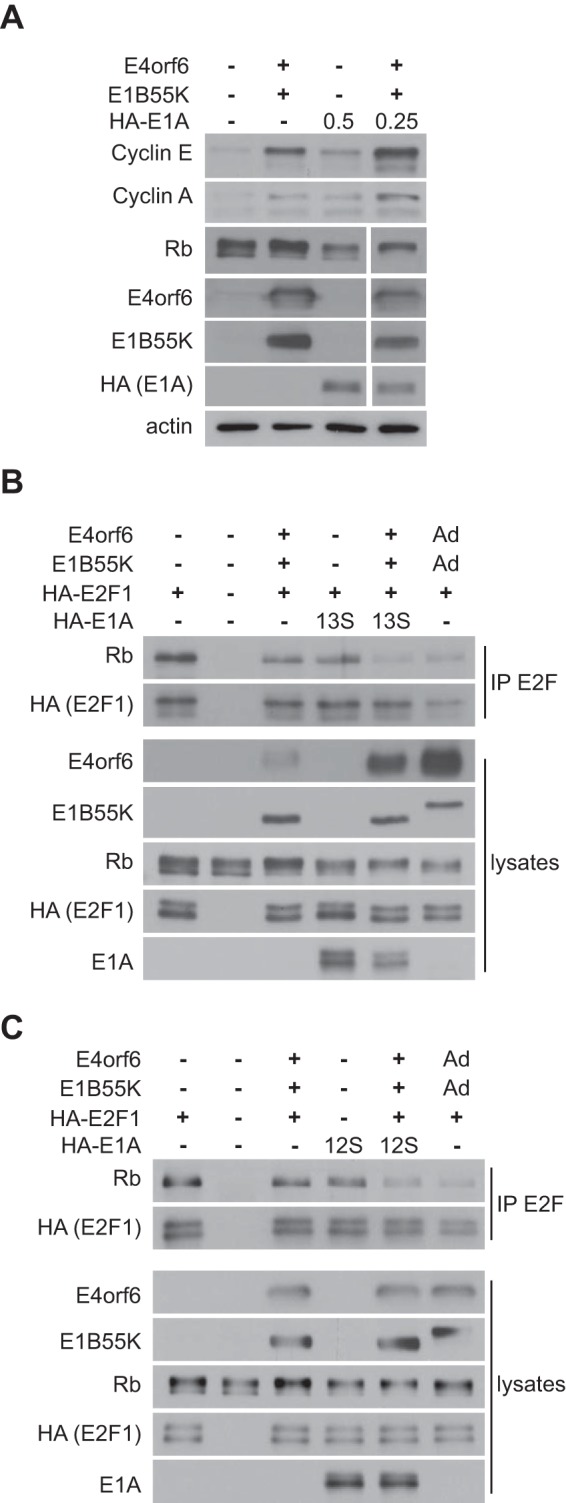
The E4orf6/E1B55K complex cooperates with E1A in inducing E2F. (A) H1299 cells were transfected with plasmid DNAs expressing E4orf6, E1B55K, and HA-13S E1A proteins as indicated, and at 48 h, whole-cell extracts were immunoblotted as indicated using the appropriate antibodies. The numbers listed at the top for HA-E1A indicate the amount of plasmid DNA used in micrograms. (B and C) H1299 cells were transfected with plasmid DNAs expressing E4orf6, E1B55K, E2F1, and 13S E1A (B) or 12S E1A (C) proteins as indicated or infected with AdE4orf6 and AdE1B55K at an MOI of 35 each (last lane). At 24 h, immunoprecipitates (IP) obtained using E2F1 antibodies as well as whole-cell extracts were immunoblotted as indicated using the appropriate antibodies.

In addition to effects on E1A protein levels, we wondered if the interactions of E4orf6 protein with E2F and E1A products might enhance the ability of E1A to activate E2F and promote viral replication. We first examined effects on Rb phosphorylation and expression of two products of E2F-regulated genes. It is important to note that to address this question, it was essential to conduct studies using equal amounts of E1A products, as expression of the E4orf6/E1B55K complex augments E1A levels. Thus, we utilized the protocol shown in Fig. 2A in which we reduced the level of plasmid DNA encoding E1A products in the presence of E4orf6 and E1B55K. Figure 4A shows that this manipulation yielded relatively similar levels of E1A 13S/289R in the presence and absence of E4orf6/E1B55K. Figure 4A also shows that expression of E1A induced a significant shift in Rb gel migration; however, this effect was considerably enhanced when E1A was coexpressed with E4orf6 and E1B55K. Figure 4A also shows by Western blotting using the appropriate antibodies that expression of either E4orf6/E1B55K or E1A 13S/289R enhanced expression of the E2F-regulated genes encoding cyclins E and A, but even higher levels were evident when all were coexpressed. These results suggested that coexpression of the ligase complex is able to enhance significantly the levels of both Rb phosphorylation associated with activation of E2F and expression of products of two E2F-regulated genes above those obtained with E1A alone. We also examined the ability of E1A products to disrupt E2F/Rb complexes. Figures 4B (13S) and C (12S) show results obtained when hemagglutinin (HA)-E2F1 was expressed in the presence or absence of E4orf6/E1B55K and 13S or 12S E1A proteins expressed from plasmid DNAs, after which extracts were immunoprecipitated with anti-E2F1 antibodies and then analyzed by Western blotting using either anti-Rb or anti-HA antibodies. Control Western blotting analyses of total cell extracts indicated the levels of all products. The results indicated that although expression of either E4orf6/E1B55K or E1A proteins reduced the level of Rb associated with HA-E2F1, expression of all enhanced this effect greatly, reaching levels comparable to those seen during infection with viral vectors expressing E4orf6 and E1B55K (lane to the right of each panel) and as found previously by many groups in wild-type virus infection (33, 37–39). (Note that E1B55K protein expressed from the viral vector contains a 6×His and heart muscle kinase site (HMK) tag, thus explaining why it migrated more slowly.)

### E4orf6 and E1B55K enhance E1A effects on viral replication.

**FIG 5:**
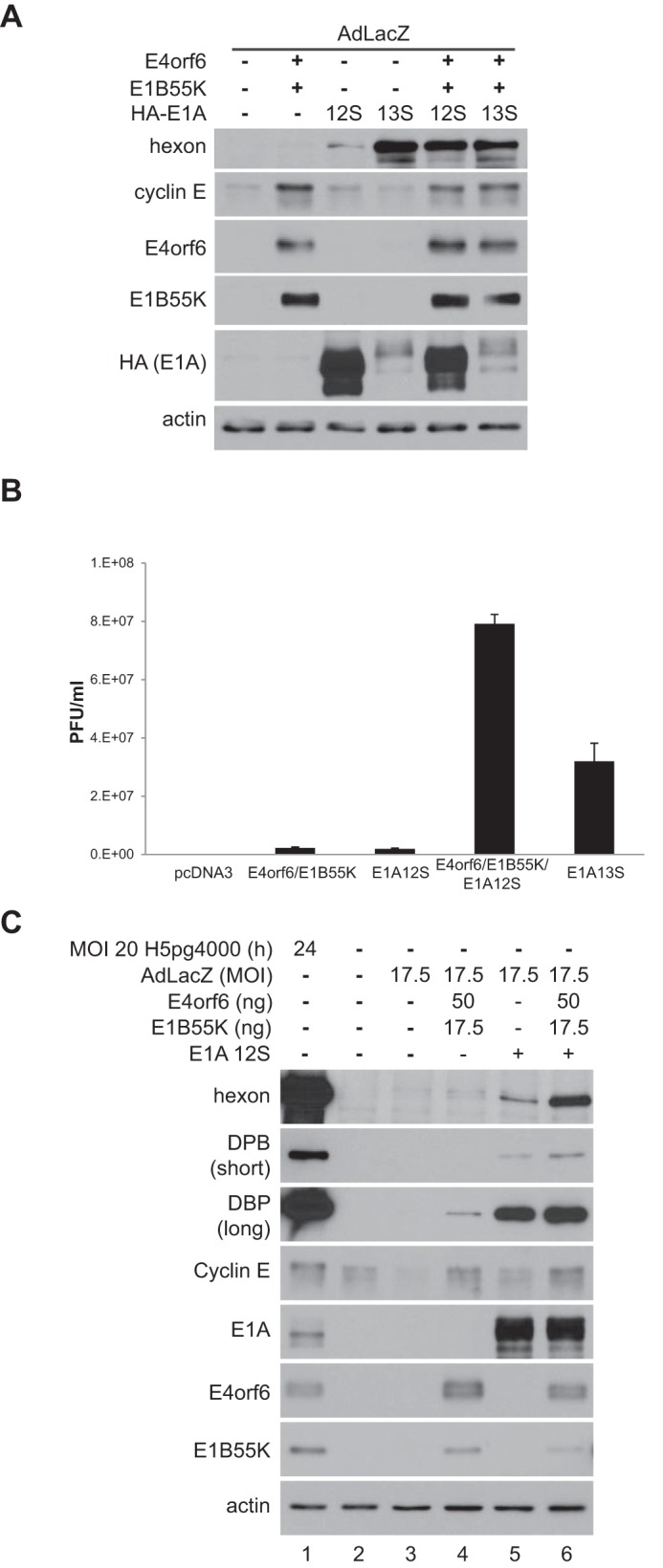
The E4orf6/E1B55K complex cooperates with E1A in inducing viral replication. (A) H1299 cells were transfected with plasmid DNAs expressing E4orf6, E1B55K, and E1A proteins as indicated and infected with AdLacZ at an MOI of 70. At 48 h, whole-cell extracts were immunoblotted as indicated using the appropriate antibodies. (B) H1299 cells were transfected with plasmid DNAs expressing E4orf6, E1B55K, and E1A proteins as indicated and infected with AdLacZ at an MOI of 70. At 48 hpi, progeny virions were harvested and reinfected onto 293 cells for plaque assay. A representative experiment is shown involving plaque assays done in triplicate. (C) H1299 cells were transfected with small amounts of plasmid DNAs expressing E4orf6 and E1B55K and infected with AdLacZ at an MOI of 17.5 for 48 h or infected with wild-type virus at an MOI of 20 for 24 h. Whole-cell extracts were immunoblotted as indicated using the appropriate antibodies.

We also determined if coexpression of E4orf6/E1B55K with E1A products could enhance the viral life cycle. Figure 5A shows that expression of only E4orf6 and E1B55K in combination with control AdLacZ viral vector infection resulted in some expression of late proteins, as shown previously in reference 31. Expression of only 12S E1A also resulted in a low level of late protein expression. Interestingly, the combination of 12S E1A with E4orf6/E1B55K resulted in much higher levels of late protein expression, demonstrating a synergistic effect. Expression of 13S E1A alone was sufficient to induce high expression of late proteins and was not increased by expression of E4orf6 and E1B55K. Also of interest, cyclin E levels correlated much more with the expression of E4orf6 and E1B55K than with either form of E1A. We have observed previously in a time course experiment (data not shown) that in replicating cells, cyclin E expression first increases and then decreases. Thus, it was not surprising in this experiment, in which replication occurred (expression of both E4orf6 and E1B55K and infection with AdLacZ vector), that there was less cyclin E than would be expected due to a synergy effect, since that time point was past the peak expression of cyclin E. Figure 5B shows that a similar synergy was seen for the production of progeny virions. H1299 cells were infected with AdLacZ and cotransfected with plasmid DNAs expressing E1A 12S/243R, E4orf6, E1B55K, or all or with plasmid DNA expressing E1A 13S/289R, and after 48 h cell extracts were subjected to plaque assays on 293 cells, as described in Materials and Methods. Whereas expression of either E4orf6/E1B55K or E1A 12S/243R led to low levels of replication of AdLacZ vector, their coexpression led to production of high levels of virus, higher even than those observed with expression of E1A 13S/289R alone. Finally, we returned to the concern that effects of the ligase complex to enhance E1A activity might result from the high levels of expression of E4orf6/E1B55K. Thus, a study was conducted in which these species were expressed at low levels from plasmid DNAs along with 12S E1A protein in cells infected at an MOI of 17.5 by the AdLacZ vector. Although in our previous studies we examined effects on hyperphosphorylation of Rb and separation of the E2F-Rb complex, assays measuring these effects were not sufficiently quantitative to determine if the E4orf6/E1B55K complex results in a partial enhancement of E1A activity. Thus, we examined by Western blotting the effects on production of late hexon protein, cyclin E, and DBP, which is produced as a result of activation of E2F. Figure 5C shows that the levels of E4orf6 and E1B55K proteins (lanes 4 and 6) following transfection were quite similar to those present in cells at 24 h following infection by wild-type virus (lane 1). Expression of both 12S E1A and E4orf6/E1B55K induced an increase in the amount of hexon from the AdLacZ genome (lanes 5 and 4); however, a larger increase was observed when all proteins were expressed (lane 6). A similar but smaller increase was also seen for the expression of E2F-inducible DBP (short exposure). The increase in the level of DBP induced by E4orf6 and E1B55K expression can be seen in the longer exposure. Interestingly, 12S E1A induced only a small amount of cyclin E (lane 5), whereas expression of E4orf6/E1B55K in the presence (lane 6) or absence (lane 5) of E1A yielded considerably more, suggesting that the ligase complex plays a significant role in inducing expression of at least this E2F-dependent species (discussed more in the Discussion). Thus, in total our findings indicated that in addition to other known functions in viral replication, the E4orf6/E1B55K complex plays an important and previously undescribed role in enhancing the effects of E1A products on events of the virus life cycle.

## DISCUSSION

In the present report, we have extended our recent findings that the Ad5 E4orf6/E1B55K complex displays an E1A-like ability to activate transcription factor E2F to promote viral replication. We demonstrated that this ability is not simply the result of overexpression of these viral proteins, and provided evidence that, via at least two mechanisms, the E4orf6/E1B55K complex may enhance the ability of E1A products to sustain an efficient replicative cycle. E1A is clearly the critical initiating factor in the activation of E2F; however, our results suggested the possibility that following the activation of expression of early viral products by E1A, newly formed E4orf6/E1B55K complexes may function to amplify and sustain these initial effects of E1A to maximize viral replication.

The E4orf6/E1B55K complex may enhance E1A activity in at least two ways. It was shown here to increase the half-life of E1A products, and in both plasmid-based assays and in infected cells, to significantly augment the levels of E1A products to better elicit their effects. As discussed above, this effect may largely be due to interactions of the E4orf6 protein with E1A products, and preliminary results with E1A mutant proteins shown in Fig. 3C suggested that such stabilization may result at least in part from the association of E4orf6 with an N-terminal region of E1A products involved in interactions with the 19S proteasomal proteins. Such interactions could inhibit or slow E1A protein degradation to increase half-life.

Also of great interest were the effects of the E4orf6/E1B55K complex on the activation of E2F transcription factors and their role in inducing early viral gene expression and DNA synthesis. Our findings indicated that coexpression of E1A products with a functional E4orf6/E1B55K ligase complex enhanced the hyperphosphorylation of Rb and separation of the inhibitory E2F/Rb complex, leading to higher levels of expression of E2F-responsive genes. While some of these studies were conducted at E4orf6/E1B55K levels in excess of those normally present in virus-infected cells, significant effects were also observed at low levels similar to those seen in infection. The clear ability of the complex at low levels to enhance the E2F-dependent expression of cyclin E and DBP (Fig. 5C) supports the idea that the E4orf6/E1B55K complex may significantly enhance the ability of E1A products to activate E2F. The extent of this enhancement and its importance in optimizing wild-type adenovirus replication will require additional studies. It is also still unclear how the E4orf6/E1B55K complex might enhance the ability of E1A products to carry out these activities. We found that E4orf6 protein interacts with both E2F and E1A products, and thus such interactions may enhance complex dissociation induced by the well-known interaction between E1A proteins and Rb family members (40–42); however, to enhance E1A effects on E2F, a functional ligase complex is required, suggesting that this process involves more than enhanced interactions with the E2F/Rb complex.

At first glance it may be surprising that E1A had such a small effect on the separation of Rb/E2F1 complexes (Fig. 4B and C); however, it has been shown that this interaction is difficult to disrupt by E1A due to an additional specific interaction of E2F1 with Rb that does not exist with other E2F species (43). Since E4orf6/E1B55K can disrupt E2F/Rb complexes for E2F1 to -3, our findings may suggest that the mechanism for separation of E2F/Rb complexes is different from that with E1A but may also potentially be complementary.

As a final note, it is interesting to speculate on the role that this E1A-like function might have in the evolution of adenoviruses. It is unknown if the E1A-like properties reported here are shared by E4orf6/E1B55K complexes of other human serotypes or species or in fact other adenoviruses. Genomic analyses of animal adenoviruses indicated the coevolution of sequences encoding E4orf6- and E1B55K-like proteins. In addition to the *Mastadenovirus* genus (which includes all human adenoviruses), E4orf6- and E1B55K-like sequences are present in the *Atadenovirus* genus, which lacks any E1A-like sequences (http://www.vmri.hu/~harrach/AdVtaxlong.htm). These findings suggested the possibility that the E1A-like function of the E4orf6/E1B55K complex could represent the original capacity of more primitive adenoviruses to replicate in terminally differentiated target cells. Of course, this faculty was greatly enhanced with the evolution of E1A proteins, which evolved with a much greater capacity to induce early viral gene expression and activate E2F. Clearly more research will be required to address these and other questions related to this new role for the E4orf6/E1B55K complex.

## MATERIALS AND METHODS

### Cell lines, plasmids, and viruses.

Human non-small-cell lung carcinoma H1299 cells (ATCC CRL-5803) and embryonic kidney 293 cells (HEK-293; ATCC CRL-1573) were grown in Dulbecco’s modified Eagle’s medium (Gibco) without antibiotics and supplemented with 10% fetal bovine serum (Multicell) at 37°C in 5% CO_2_. The plasmids used in the studies were pcDNA3-E4orf6 (44), pcDNA3-E1B55K (45), pcDNA3-HA-E1A-12S and pCDNA3-HA-E1A-13S (46), and pcDNA3-HA-E2F1 (kindly provided by Doron Ginsberg). The adenoviral vectors and virus mutants used have been described previously: AdE1B55K (47, 48), AdE4orf6 (47, 48), AdLacZ (49), H5*pg*4100 (WT) (50), H5*pm*4154 (E4orf6^−^) (51), H5*pm*4149 (E1B55K^−^) (52), and the *dl*309 (53) and E1A *dl*1101, *dl*1102, and *dl*1504 mutant (54) viruses.

### Antibodies and reagents.

The rabbit polyclonal antibodies used were E4orf6 (1807) (55) and Ad5 capsid (56). The mouse monoclonal antibodies used were as follows: Ad5 E1B55K 2A6 (57), E2A DNA-binding protein B6-8 (58), actin C4 (catalog no. 691001; MP Biomedicals), Rb OP66 (catalog no. OP66; Millipore) or 4H1 (catalog no. 9309; Cell Signaling), cyclin A BF683 (catalog no. Sc-239; Santa Cruz), HA tag HA.11 clone 16B12 (catalog no. MMS-101R; Covance), E2F1 KH95 (catalog no. Sc-251; Santa Cruz), Ad5 E1A M58 (from Roger Grand), and M73 (59), cyclin E HE12 (catalog no. sc-241; Santa Cruz). The rat monoclonal antibody was E1B55K 4E8 (52). The horseradish peroxidase (HRP)-conjugated secondary antibodies for detection in Western blotting were goat anti-mouse IgG, goat anti-rabbit IgG, and goat anti-rat IgG (Jackson ImmunoResearch Laboratories) and rat anti-mouse κ light-chain-specific clone 187.1 (catalog no. 559751; BD Biosciences). Additional reagents included Lipofectamine 2000 reagent (Invitrogen) and cycloheximide (catalog no. CYC003.5; BioShop).

### DNA transfections and infections.

DNA transfections were done using Lipofectamine 2000 reagent according to the manufacturer’s recommendations. The final amounts of DNA per well were equalized by addition of vector plasmid pcDNA3. For infections, cells infected with viruses diluted in infection medium (0.2 mM CaCl_2_, 0.2 mM MgCl_2_, and 2% serum in phosphate-buffered saline [PBS]) for 90 min before removal and the addition of normal growth medium. For most experiments, a multiplicity of infection (MOI) of 35 PFU per cell was used for viral vectors, except where indicated, and the final amounts of vectors were adjusted to be equal by the addition of AdLacZ. Infections with WT and mutant viruses were done at 5 focus-forming units (FFU)/cell for coimmunoprecipitation (co-IP) experiments and 20 FFU/cell for infections in low-expression experiments. Infections with the *d*l309, *dl*1101, *dl*1102, and *dl*1504 mutants were done at an MOI of 20 PFU/cells. For infections and cotransfections, cells were first infected at the indicated MOI for 90 min and then transfected with the indicated plasmid DNAs. Infection for the time course experiment was done in normal medium at an MOI of 5 FFU per cells, and cells were harvested at 4-h intervals from 8 h p.i. to 24 h p.i.

### Protein extraction.

Cells were washed with PBS and removed at different times postinfection/posttransfection by incubation for 5 min with gentle agitation with 0.53 mM EDTA and collected by centrifugation. For viral DNA amplification of extracts and other protein blotting, cells were lysed for 20 min on ice in the lysis buffer (50 mM Tris-HCl [pH 8.0], 150 mM NaCl, 5 mM EDTA, 1% NP-40, 0.1% SDS, 0.1% Triton X-100, protease inhibitor cocktail [Sigma], 1 mM Na_3_VO_4_, 10 mM NaPPi, 10 mM NaF) and clarified by centrifugation at 13,000 × *g* for 10 min. For immunoprecipitation studies, cells were lysed for 20 min on ice in lysis buffer (20 mM Tris-HCl [pH 7.5], 150 mM NaCl, 2 mM EDTA, 1% Triton X-100, 5% glycerol, protease inhibitor cocktail [Sigma], 1 mM Na_3_VO_4_, 10 mM NaPPi, 10 mM NaF) and clarified by centrifugation at 13,000 × *g* for 10 min.

### Immunoblotting.

Equal amounts of proteins were separated by SDS-PAGE and then transferred to polyvinylidene difluoride membranes (Millipore) blocked using 5% skimmed milk prior to antibody blotting. Primary antibodies were added to the membranes for 2 to 3 h at room temperature or overnight at 4°C. Membranes were washed with PBS containing 0.1% Tween 20, and the secondary antibody was added for 1 h at room temperature. Detection was performed using Western Lightning Chemiluminescence Reagent Plus (PerkinElmer).

### Viral DNA amplification.

H1299 cells were infected with Ad vectors expressing the appropriate combination of E4orf6 and E1B55K or AdLacZ vector at the indicated MOI. Cell extracts were made as described above and treated with proteinase K for 1 h at 55°C. Viral DNA content was measured by PCR with primers specific for the Ad5 fiber gene (forward, CACCCCTCACAGTTACCTCAGAAGCCC, and reverse, GTCTGTTTTGAGAATCAATCCTTAGTCCTC). PCR products were visualized on 1.2% agarose gels. Purified adenovirus DNA was used as a positive control for PCR analyses.

### Measurement of progeny virions.

H1299 cells were infected with AdLacZ vector at an MOI of 35 and cotransfected with the indicated plasmids for 48 h. Cells were pelleted, lysed by 4 cycles of freezing and thawing, and clarified by centrifugation at 1,900 × *g* for 10 min. Virus titers in the supernatant were determined by plaque assay in HEK293 cells. A representative experiment is shown, with error bars from an average of three independent plaque assays.

### Immunoprecipitations.

Cells were transfected or infected as indicated for each experiment. Between 200 and 500 µg of proteins was precleared overnight at 4°C with protein G Sepharose beads (GenScript). Beads were removed by centrifugation, and supernatants were incubated with either anti-E4orf6 polyclonal antibody 1807, anti-E1A mouse monoclonal antibody, or anti-E2F1 KH95 mouse monoclonal antibody, followed by incubation with protein G Sepharose (GenScript). The beads were extensively washed in lysis buffer and examined by SDS-PAGE. The detection of Rb and HA-tagged proteins in Western blots with primary mouse monoclonal antibodies was done with secondary HRP-conjugated rat anti-mouse κ light-chain-specific antibody.

### Cycloheximide treatment.

H1299 cells were transfected with indicated plasmids for 43 h at 37°C. The medium was replaced by fresh medium containing 25 μg/ml cycloheximide, cells were harvested at the indicated time points, and protein extracts were made as indicated above. Samples were separated by SDS-PAGE, transferred to polyvinylidene difluoride membranes, and blotted as described earlier. Autoradiography films were scanned, and the HA-E1A band was analyzed and quantitated using ImageJ 1.42q software (NIH, United States). Each condition was normalized to the respective 0-h time point, and error bars were from three independent experiments.
